# Is a (satellite) image worth a thousand data points? Comparing machine learning approaches to predict environmental and social inequalities in England

**DOI:** 10.1371/journal.pone.0356472

**Published:** 2026-08-21

**Authors:** Barbara Metzler, Martin Fleischmann, Daniel Arribas-Bel

**Affiliations:** 1 Geographic Data Science Lab, University of Liverpool, Liverpool, United Kingdom; 2 The Alan Turing Institute, London, United Kingdom; 3 Department of Social Geography and Regional Development, Charles University, Prague, Czechia; University of Ferrara: Universita degli Studi di Ferrara, ITALY

## Abstract

Accurately mapping environmental and social inequalities at fine spatial scales is critical for urban policy, yet the data required are often costly and infrequently updated. Vision foundation models can extract information directly from satellite imagery, offering a rapid and scalable alternative. We compare three modelling pipelines for predicting two contrasting indicators of urban inequality – air pollution and house prices – across England on a fine hexagonal grid: regression models trained on structured *features of form and function* (census, land cover and urban morphology), models trained on 128-dimensional image embeddings from a geospatial foundation model and a hybrid of the two, each evaluated with and without coarse regional context. Structured features achieve the best overall accuracy, but image embeddings become competitive once regional context is added – most clearly for air pollution, where image-only models reach a median R^2^ of 0.78 (0.85 with regional context), indicating that the embeddings capture genuine image signal. For house prices the picture is more cautious: image-only models achieve a median R^2^ of around 0.58; however, much of the embeddings’ apparent gain reflects coarse spatial location rather than image content. Off-the-shelf satellite embeddings, while not yet surpassing data-intensive approaches, are a promising low-cost complement, particularly for rapid, large-scale, or repeated analyses and in settings where traditional data are limited.

## Introduction

Geospatial data on ground measurements, such as environmental and social inequalities, are crucial for informed decision-making and the development of effective policies. These measurements guide local and national governments in allocating resources and serve as the foundation for efforts to monitor and improve human livelihoods, particularly within urban contexts. Cities, where most people live and spend the majority of their time [1], often exhibit growing inequalities with far-reaching impacts. Large cities like London, for instance, display pronounced differences in living environments and health outcomes [2–4]. Such disparities also persist at the broader regional scale, with the United Kingdom (UK) notably identified among OECD countries as exhibiting significant regional inequalities. Socioeconomic gaps, particularly between the prosperous south-east and the rest of the UK, have continued to widen in recent decades [5–7].

Understanding and accurately measuring these inequalities is central to the creation, monitoring, and evaluation of policies intended to reduce geographic disparities. Considerable attention has been dedicated to comprehensively documenting and addressing persistent regional and intra-urban inequalities in the UK [8]. However, effectively monitoring changes in inequalities across multiple scales, from neighbourhoods and individual cities to entire nations, demands high-resolution spatial and temporal data. This requirement presents a significant challenge: many key datasets, such as national censuses, travel surveys, and infrastructure maps, are updated infrequently and on timelines that rarely align [9]. Additionally, the creation and maintenance of these traditional datasets remain costly and resource-intensive, limiting their timely availability for policy decisions.

The need for frequent, wide-scale, and up-to-date urban data has grown substantially, driven by initiatives like *Digital Twin* models designed to simulate complex city dynamics in near real-time [10,11]. Tools for urban and land-use planning increasingly rely on detailed and current information to track inequalities and evaluate the potential impacts of policy interventions [12,13]. Recent methodological advancements have enabled the creation of spatially and temporally consistent datasets, enhancing our capacity to analyse complex demographic and socioeconomic changes at high resolutions [8,13–15].

Recent advancements in satellite imagery and machine learning have opened new avenues for overcoming the data challenges described above. Satellite images capture a wide range of information across varying scales and at flexible time intervals, enabling detailed analyses of individual buildings as well as broad studies spanning entire continents. Automated image-processing methods further enhance the capacity to rapidly infer insights, making satellite imagery a valuable source of information to guide decision-making [16]. By leveraging visual and spectral data, satellite images can be used to infer on-the-ground conditions such as land use, urban development, population density, and environmental features [17–21]. Machine learning can help to transform pixel-level data into actionable information, reducing reliance on costly, labour-intensive, and ad hoc data collection. Nonetheless, translating high-dimensional satellite data into meaningful metrics remains complex, requiring interpretation of abstract image representations. Moreover, many satellite-based models are highly specialised, often necessitating extensive fine-tuning to adapt to new tasks or contexts.

The emergence of foundation models in geospatial artificial intelligence provides a more flexible alternative. Unlike traditional models tailored narrowly to specific tasks, foundation models are pre-trained on large, diverse datasets, enabling adaptation to multiple downstream applications with minimal additional training [22–24]. This scalability is particularly valuable in remote sensing, where data and associated applications are inherently complex and heterogeneous. Foundation models integrate multi-spectral and multi-temporal imagery effectively, capturing intricate patterns and offering more generalisable representations for a wide variety of tasks [24,25].

Recent research demonstrates that foundation models often outperform traditional task-specific approaches, especially when labelled data are scarce [26–28]. Transformer-based architectures, pre-trained on large multispectral satellite image datasets, have successfully addressed diverse Earth Observation (EO) tasks ranging from cloud detection and object identification to multi-temporal segmentation [23,29,30]. The availability of pre-trained embeddings greatly simplifies deployment, reducing the technical barriers associated with implementing sophisticated machine learning workflows. Critically, the use of embeddings also eliminates the need for extensive fine-tuning of the underlying neural architectures, substantially reducing computational complexity and facilitating easier implementation of predictive tasks in practice.

With increasing availability of high-quality embeddings derived from foundation models [23,31,32], there is a need to evaluate their performance systematically against established geospatial datasets. Such an assessment is particularly crucial given the expanding use of satellite imagery in urban planning and policy-making contexts, where frequent updates and comprehensive spatial coverage are vital. However, the adoption of these innovative methods must carefully consider practical constraints such as computational resources and data availability, especially in settings with limited analytical capacity or budgetary restrictions.

This paper makes two key contributions. First, it compares the performance of models trained on off-the-shelf satellite image embeddings with those relying on traditional data sources describing urban form and function. Second, it evaluates and reports the complexity of data preparation, computational costs, and performance trade-offs for each approach. We frame this throughout as a comparison of practical, end-to-end pipelines, rather than a controlled test of the raw representational capacity of the two data types. These insights are valuable for researchers and policymakers, especially those operating under resource constraints, as they aim to balance data quality, computational efficiency, and model accuracy.

### Overview of approach

Our analysis systematically compares models using satellite image embeddings with those relying on multiple geospatial datasets describing urban form and function. These traditional datasets, derived from censuses, surveys, and building data (Materials and Methods), are collectively referred to as ‘features of form and function’ (FFF) throughout this paper.

We focus on predicting two critical indicators representing distinct yet complementary dimensions of urban inequality: air pollution and house prices. Air pollution provides a clear environmental indicator closely tied to physical attributes such as pollution sources, land use patterns, and infrastructural elements like roads. While air pollution itself may not be directly visible from satellite imagery, these underlying determinants frequently are. Previous studies have successfully employed deep learning with satellite imagery in similar contexts, especially where traditional data sources were scarce or unavailable [22,33–35].

House prices, in contrast, capture socioeconomic dimensions influenced by complex social factors, including wealth distribution, neighbourhood desirability, and accessibility to amenities. Although these attributes are typically less directly observable from overhead images, prior research using night-time lights and street-level imagery has identified visual cues, such as building conditions and visible signs of deprivation, that correlate with socioeconomic outcomes [36–39]. Together, air pollution and house prices offer a holistic perspective on urban inequalities, encompassing both environmental and socioeconomic aspects. We selected these two outcomes to span the visibility spectrum of urban-inequality indicators: air pollution has physically observable determinants (land use, road networks, industrial sources) that are plausibly captured in satellite imagery, whereas house prices reflect largely latent socioeconomic dynamics that are not fully observable in satellite imagery. This contrast allows us to assess how model performance differs across outcomes with determinants that vary in their observability from satellite imagery. These two indicators were also among those prioritised through co-development with local-government partners on land-use decision-making, underscoring their practical relevance for urban planning.

To predict these indicators, we apply a robust and widely used machine learning algorithm (XGBoost), due to its proven effectiveness in capturing complex relationships within large datasets. Full details of the model setup, including hyperparameter tuning, are provided in the Methods section.

**Fig 1:**
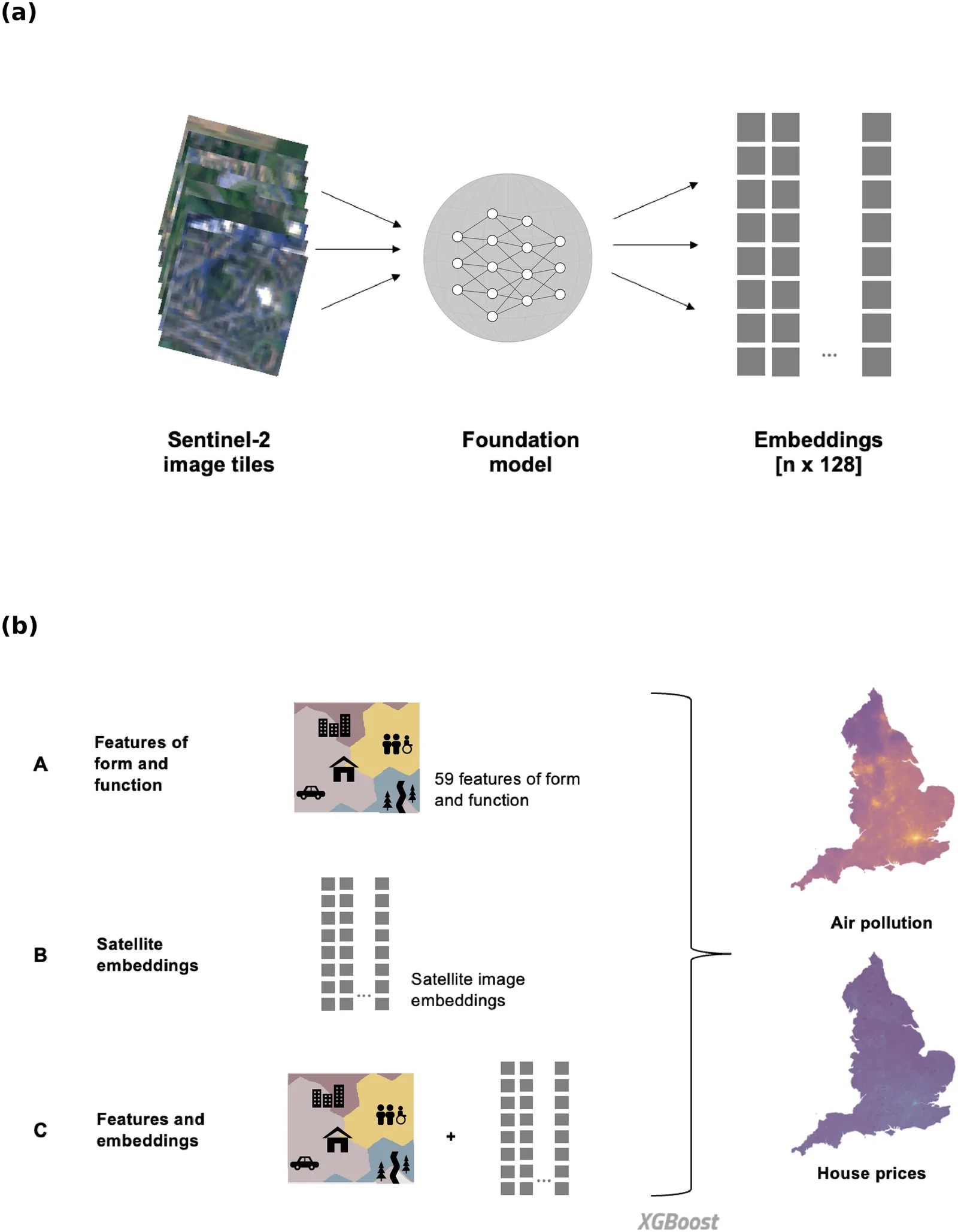
Overview of the embedding pipeline and the three modelling approaches compared. (a) Sentinel-2 tiles are processed through a vision-foundation model to generate embeddings of dimension [n × 128]. (b) We compare three supervised learning strategies for two target variables (air pollution and house prices): a tabular baseline using census, survey and urban morphology features (features of form and function); an image-only model trained solely on the embeddings; and a hybrid approach that concatenates embeddings with features of form and function. Performance is evaluated under five-fold spatial cross-validation. The image tiles in panel (a) contain modified Copernicus Sentinel-2 data (2017–2018) from the global cloud-free composite of Corbane et al. [40].

We evaluate three distinct modelling approaches (illustrated in Fig 1), each differing in data requirements, computational complexity, and preparation time:

- **Approach A** uses structured geospatial features (FFF), including population density, census data, land-use types, and building morphology, all widely adopted in urban planning and geospatial modelling contexts.
- **Approach B** employs advanced vision transformers, specifically leveraging satellite image embeddings obtained from pre-trained foundation models. These embeddings encapsulate abstract yet powerful visual representations extracted directly from satellite images (Fig 1). Crucially, using these embeddings removes the need for extensive fine-tuning, thereby simplifying model implementation and significantly reducing computational complexity.
- **Approach C** combines structured FFF data with satellite image embeddings, integrating established domain-specific insights with rich visual features extracted from imagery. This hybrid method offers a comprehensive analysis, clarifying the additional predictive power gained over using either traditional structured data (Approach A) or image embeddings alone (Approach B).

Additionally, we evaluate the impact of incorporating regional context by including spatial indices derived from the H3 hexagonal spatial grid at resolution 3 (S3 Fig). Incorporating such regional context is standard practice in geospatial modelling, as it helps account for broad spatial trends that might otherwise obscure or bias local predictions. To quantify how much predictive power stems from this coarse spatial context alone, we additionally evaluate a region-only baseline that uses only the H3 resolution-3 parent-cell location as input (see the Results).

By comparing these three approaches systematically, we aim to determine whether unstructured data derived from emerging satellite-based techniques can offer competitive predictive accuracy relative to traditional structured datasets. This assessment explicitly evaluates whether foundation models, by reducing data collection costs and computational barriers, can feasibly expand the scope of urban analyses. This evaluation informs the trade-offs between cutting-edge methods and constraints related to data availability, computational resources, and interpretability.

To further inform this comparison, key metrics of Approaches A and B, such as computational complexity, processing time and interpretability, are summarised in Table 1.

**Table 1:** Comparison of modelling approaches based on input data type and practical characteristics.

Metric	Features of Form and Function (FFF)	Satellite Image Embeddings
Input type	Structured tabular data	Visual (image-based) data
Data sources	4 datasets (population, census, land cover, morphometrics)	Sentinel-2 satellite composite (RGB)
Initial dimensionality	75 variables	~200 million pixels
Final input dimensionality	n×59	n×128
Spatial resolution	OA-level aggregates (~100–625 people)	10 m per pixel
Temporal resolution	5–10 years (static snapshots)	Bi-weekly potential (if cloud-free)
Data scope	England only	Global (Sentinel-2 coverage)
Preprocessing time	Several days (multi-source wrangling)	Tiling 1,382,771 chips (~700 GB intermediate tiles) + ~24 h embedding extraction
Embedding extraction	n/a	~24 h, single GPU (Swin-v2-B forward pass); HPC envelope ≤250 GB RAM / 72 h walltime
Feature-store size	~0.6 GB (1.38M × 59 parquet)	~1.3 GB (1.38M × 128 table)
Model training (England, 1.38M units)	~2 s/fold (region) to ~10 s/fold (FFF, 110 feats); full 5-fold in minutes, single consumer GPU	comparable (uses the precomputed embedding table)
Peak training memory	~2–3 GB	~2–3 GB
Skill requirements	Intermediate (data wrangling, modelling)	Intermediate (embedding use, ML pipelines)
Interpretability	High (feature-level transparency)	Low to moderate (abstract, latent features)^a^

^a^Interpretability is reduced due to the abstract nature of deep feature embeddings.

## Materials and methods

### Satellite images

We use the satellite composite dataset described by Corbane et al. [40], consisting of a global, cloud-free composite created from Sentinel-2 data (2017–2018). Images are segmented into uniform tiles, each approximately 42×42 pixels. As a preprocessing step, we apply a hexagonal H3 masking procedure at resolution 9 [41], consistent with the spatial scale of our analysis. To ensure spatial consistency and prevent potential geographical bias, individual masks are aggregated into a uniform mask, applied consistently across all tiles (S1 Fig).

### Features of form and function (FFF)

Our structured dataset (FFF) comprises 59 predictor variables derived from four principal sources, capturing dimensions of land cover, population density, urban form, function, and morphology [42]. Mid-2020 population data at Output Area (OA) level were obtained from the UK’s Office for National Statistics (ONS), ensuring temporal alignment with other datasets (2018–2020). Workplace population data, based on the 2011 Census, were categorised by industry groups, and land cover proportions were derived from the CORINE Land Cover 2018 classification. Initially, 75 variables were identified, with highly correlated pairs removed to improve model interpretability.

### Outcome variables

We modelled two inequality indicators, air pollution and house prices, representing environmental and socioeconomic dimensions, respectively.

The air pollution measure combines PM2.5, PM10, *NO*_2_, *O*_3_ and *SO*_2_ concentrations, sourced from the UK AIR dataset by DEFRA (https://uk-air.defra.gov.uk/data/gis-licences) at a 1 km grid resolution. We combine the five regulated pollutants into a single weighted scalar, following standard European Environment Agency practice, so that the outcome is one interpretable, policy-aligned air-quality index rather than five separately modelled pollutants:


$$\begin{array}{c} Q_{air}=\frac{PM2.5}{1}+\frac{PM10}{2}+\frac{NO_{2}}{4}+\frac{O_{3}}{5}+\frac{SO_{2}}{10} \end{array}$$

*O*_3_ data were based on days exceeding 120 µg/m^3^. UK AIR data represent model-derived rather than directly measured values, and were spatially interpolated from the original 1 km grid onto OA geometries, and subsequently averaged within the H3 hexagonal grid used in our analysis.

For house prices, we used price-per-square-metre (sqm) data derived from Chi et al.’s dataset (2018–2019) [43], which integrates Land Registry Price Paid Data with Domestic Energy Performance Certificates. This approach allowed us to accurately capture local property market variations at OA level, addressing limitations inherent in conventional property datasets. Given the temporal gap since data collection, our model results are presented as percentage changes from the mean baseline price per sqm at the OA level, rather than absolute values. We model price relative to the OA baseline because absolute prices are dominated by between-region level effects that would otherwise overwhelm local variation. We note that this transformation deliberately reduces between-region variance; this is precisely the variance recovered by the region-only baseline introduced in the Results, which is why coarse regional location alone still explains a substantial share of the transformed target.

### Foundation model and image embedding

We use the SatlasPretrain with Swin Transformer backbone model [30] to create satellite image embeddings. The Swin Transformer backbone is a hierarchical vision transformer architecture that uses shifted windows to efficiently capture both local and global contexts in images, enabling it to achieve high performance on various vision tasks while maintaining computational efficiency [44]. We selected the SatlasPretrain model due to its prior training on similar data, specifically Sentinel-2 imagery, with images of 10m resolution per pixel and with three bands (RGB). The dataset the model was trained on covers 302 million labels under 137 categories and seven label types. The embeddings include four positional encodings of different scales. The extracted feature vector is very high-dimensional (n × 2,704,256) and in order to be suitable for further machine learning methods (*n* > *p*), we apply feature pyramid network [45] across feature maps to combine coarse and fine-grained representations. This produces four final feature maps, each containing 128 channels. This dimensionality aligns with the default prediction pipeline used in the SatlasPretrain model for classification tasks. Following their standard setup, we apply average pooling to reduce these feature maps into embeddings with dimensions [n × 128]. Our approach mirrors the Satlas classification pipeline precisely, omitting only the final linear prediction layer to directly extract the embeddings for our analysis (S2 Fig). We note a methodological dependency: the SatlasPretrain backbone is itself pre-trained on Sentinel-2 imagery, so part of the embeddings’ advantage may reflect domain alignment between the pre-training and evaluation distributions. Results may therefore not transfer directly to embeddings pre-trained on other sensors or data distributions; we return to this point in the Discussion.

### Predictive model

We use an XGBoost regression model [46] to predict either air pollution or house prices across the study area. In order to capture the spatial characteristics of the indicators, we augment our dataset with an additional 59 features, representing the spatial lag of the original set. The lag is computed as the mean value of the variable within a neighbourhood, whose definition is established empirically. We report the spatial lag as results of contiguity from orders 1–5, and using both binary and inverse weighting schemes.

To ensure that no information from the training set leaks into the test data, especially given the use of spatial lags in our prediction model, we implemented a careful evaluation strategy. The data were partitioned into hexagonal regions, which were then assigned to one of five folds. For each fold, spatial lags were calculated independently within the training data. The model was trained on four of the folds and tested on the remaining fold, allowing for a thorough assessment of its performance across different subsets of the data. We assess the model’s performance using the median *R*^2^ and mean squared error across the five folds, using spatial contiguity from order 5, and using binary weighting schemes. We use the highest predicting model to report the final accuracy reported in the Results. The best model conditions, such as learning rate and number of estimators, are reported in S1 Table for the air pollution model and S2 Table for the house price model.

The identical spatial-lag construction (order-5 binary contiguity, row-standardised) and the identical hyperparameter search budget were applied to every approach (A, B and C) and to every regional-context variant, so that performance differences reflect the input features rather than the spatial-smoothing or tuning design. Spatial lags are recomputed within each fold’s training nodes only, preventing leakage across the spatial cross-validation. Performance improves smoothly and monotonically with increasing lag order, with the ranking of approaches preserved throughout. For the FFF model, median *R*^2^ increases from 0.79 to 0.82 between lag orders 1 and 3 for air pollution and from 0.50 to 0.53 for house prices, approaching the corresponding order-5 values of 0.84 and 0.56. On average, each additional lag order improves performance by approximately 0.012 *R*^2^ points. The absence of any discontinuity indicates that the results are not an artefact of the spatial-smoothing choice. We emphasise that this study compares practical end-to-end pipelines using 59 features of form and function plus their spatial lags versus a single 128-dimensional off-the-shelf foundation-model embedding, rather than performing a controlled test of raw representational capacity. The two encode different information at very different preprocessing cost.

#### Accounting for geographic region.

Significant regional trends were evident, especially in house prices across England. To account for these large-scale geographic variations, regional context was integrated by adding H3 hexagon centroids at resolution 3 (approximately 12,393 km^2^ per hexagon) to the models (S3 Fig). Each resolution-3 hexagon encompasses approximately 117,660 smaller resolution-9 hexagons, corresponding to the scale of individual satellite image inputs. Including such regional geographic coordinates helped ensure models captured broader spatial trends accurately. To isolate the predictive contribution of this coarse spatial context, we additionally fit a region-only baseline that uses only the H3 resolution-3 parent-cell centroid as input (26 regions across England), reported alongside Approaches A, B and C in the Results.

## Results

### Predictions of inequality measures

Our analysis reveals that satellite image embeddings generated by foundation models are competitive with traditional geospatial datasets across both environmental and socioeconomic indicators. Predictive performance, however, varies considerably depending on the type of inequality measured. Air pollution, an environmental measure closely tied to observable physical features, was predicted more accurately (median *R*^2^ range: 0.78–0.91) compared to house prices, a socioeconomic indicator driven by complex and less visually discernible factors (median *R*^2^ range: 0.37–0.72). Notably, incorporating regional context consistently improved predictive accuracy for both indicators (Tables 2 and 3). This improvement was especially evident in embedding-only models, highlighting that embeddings benefit significantly from spatial contextualisation. All performance figures are obtained under five-fold spatial cross-validation with folds defined by H3 resolution-6 parent cells and spatial lags recomputed within each training fold, so spatially adjacent hexes never appear in both training and test sets (Methods).

**Table 2:** Performance of air pollution model, *R*^2^ median across five folds.

Model	Without regional trends	With regional trends
Region only (H3 res-3)^a^	—	0.62
Approach A (FFF)	0.8387	0.9022
Approach B (embeddings)	0.7810	0.8547
Approach C (FFF+embeddings)	0.8651	0.9049

^a^Target predicted from the H3 resolution-3 parent-cell centroid alone (26 regions across England); median *R*^2^ over the five spatial folds. The region-only baseline has no “without regional trends” value because it is itself the regional signal.

**Table 3:** Performance of house price model, *R*^2^ median across five folds.

Model	Without regional trends	With regional trends
Region only (H3 res-3)^a^	—	0.48
Approach A (FFF)	0.5562	0.7167
Approach B (embeddings)	0.3710	0.5792
Approach C (FFF+embeddings)	0.5835	0.7045

^a^Target predicted from the H3 resolution-3 parent-cell centroid alone (26 regions across England); median *R*^2^ over the five spatial folds. The region-only baseline has no “without regional trends” value because it is itself the regional signal.

**Fig 2:**
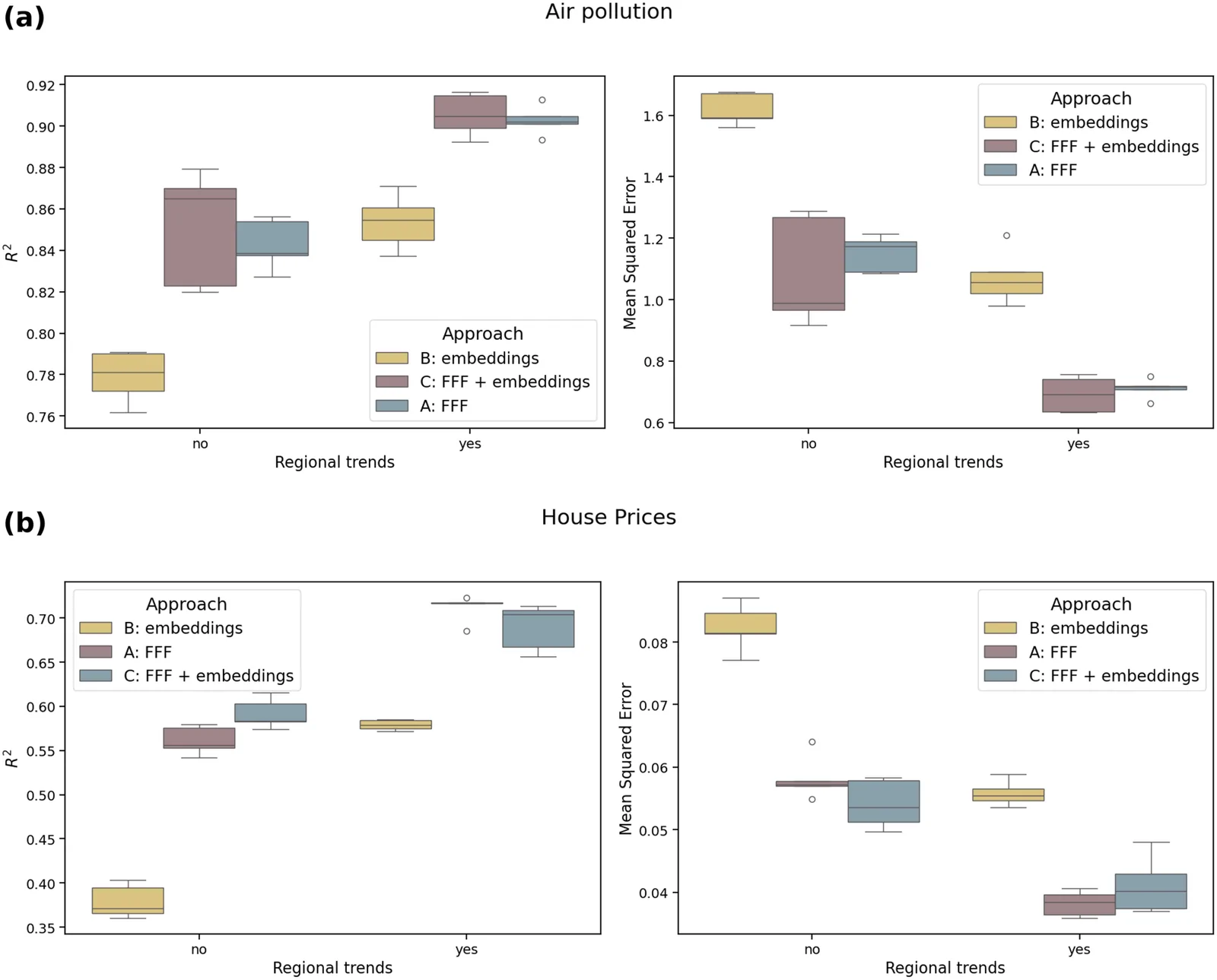
Model performance across the five spatial folds. (a) Air pollution and (b) house prices, showing *R*^2^ (left) and mean squared error (MSE, right) for each approach, with and without regional trends.

For air pollution (Fig 2 and Table 2), models using traditional features of form and function (FFF, Approach A) achieved a baseline median *R*^2^ of 0.84, improving notably to 0.90 when regional trends were included. In contrast, embedding-only models (Approach B) initially performed somewhat lower at 0.78 but improved significantly to 0.85 upon incorporating regional context, substantially narrowing the gap with traditional methods. Combining both structured data and embeddings (Approach C) produced the best overall performance, reaching a median *R*^2^ of 0.90. These results confirm that foundation model embeddings, particularly when enriched with spatial context, effectively capture critical visual cues linked to environmental factors such as land use patterns and infrastructure.

To isolate the contribution of coarse spatial location, we additionally fit a region-only baseline that predicts the target from the H3 resolution-3 parent-cell centroid alone (26 regions across England). This baseline reaches a median *R*^2^ of 0.62 for air pollution. The embedding-only model (0.78) clearly exceeds this region-only baseline, indicating that the embeddings carry image information well beyond coarse spatial location. Air pollution is therefore a target for which these foundation-model embeddings extract genuine, spatially localised signal.

**Fig 3:**
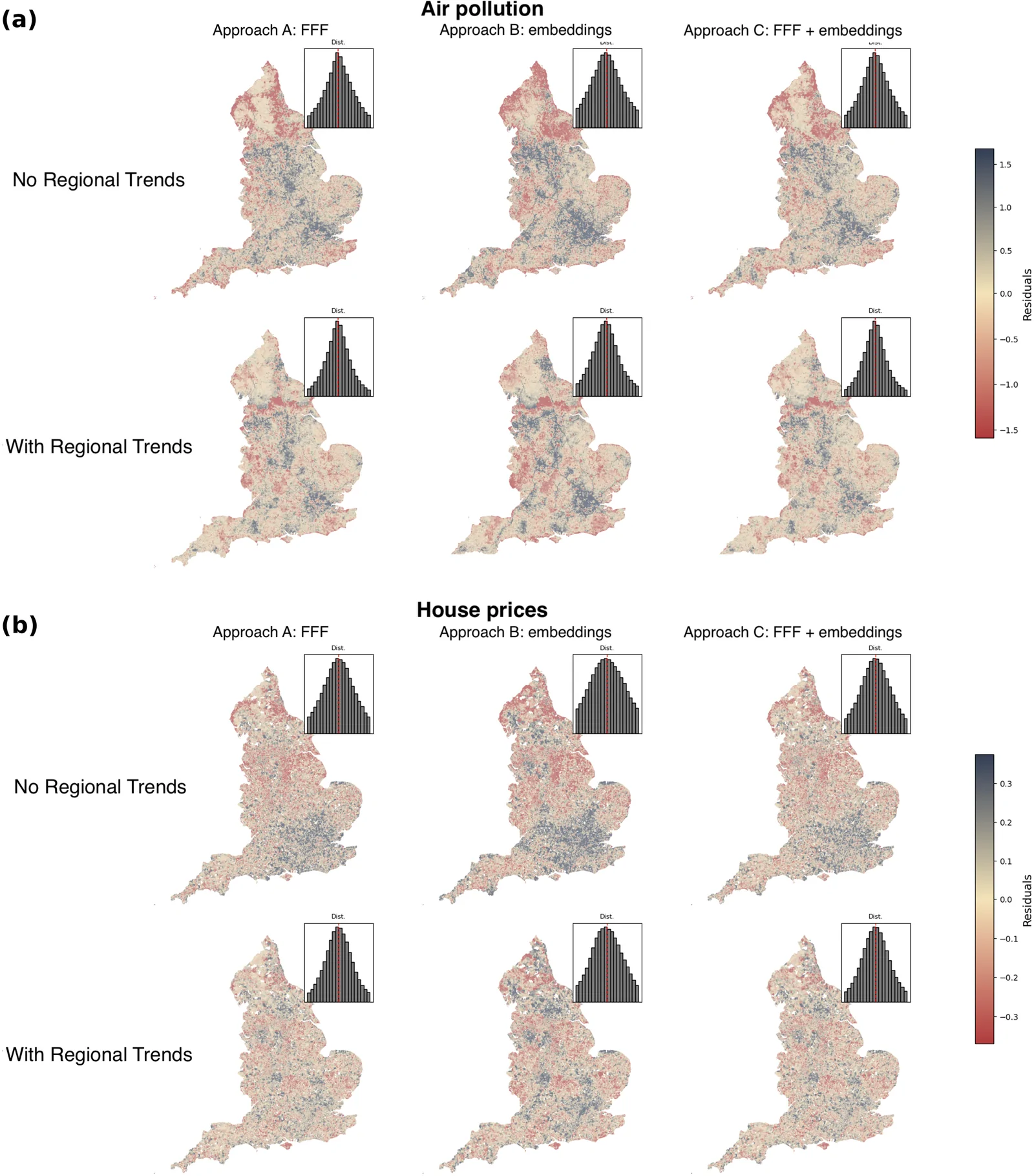
Spatial distribution of model residuals across England. (a) Air pollution and (b) house prices. Blue indicates areas of underprediction and red indicates areas of overprediction. The England outline is derived from Office for National Statistics boundary data (Contains OS data © Crown copyright and database right, Open Government Licence v3.0).

Spatially, residual maps of air pollution predictions (Fig 3) revealed systematic overestimations in northern regions when regional information was excluded. Adding regional context reduced these errors, bringing local variations into clearer focus. Notably, underpredictions from the embedding-only approach were consistently aligned with main road networks, demonstrating the embeddings’ ability to visually detect localised environmental conditions but underscoring the importance of incorporating spatial context to interpret these features more accurately.

House price predictions, however, posed greater challenges due to their inherent complexity and reliance on socioeconomic dynamics not always visible in overhead imagery (Fig 2 and Table 3). The FFF baseline (Approach A) achieved an *R*^2^ of 0.56 without regional context, while embedding-only predictions (Approach B) lagged at 0.37. Introducing regional trends substantially boosted accuracy for all methods, particularly elevating embedding-only performance to an *R*^2^ of 0.58 and the FFF baseline to 0.72. These improvements emphasise the critical role of regional context in capturing spatially dependent socioeconomic factors.

For house prices the region-only baseline tells a different and more cautionary story. Coarse location alone reaches a median *R*^2^ of 0.48, which exceeds the embedding-only model (0.37), and embedding-plus-region (0.58) improves only modestly over region alone. Much of the apparent embedding-based performance for house prices therefore reflects coarse spatial position rather than image content. Notably, once regional context is present the embeddings add essentially nothing: the full hybrid (FFF + embeddings + region, 0.70) does not exceed FFF + region (0.72). This may reflect the limited visibility of key house-price determinants in Sentinel-2 imagery, limitations in the information captured by the embeddings, or a combination of both.

Spatial analyses of house price residuals (Fig 3) revealed consistent underpredictions in major urban centres such as London, as well as in coastal regions like Norfolk and Cornwall. These patterns reflect the limits of using only visual and structural indicators from satellite imagery to fully capture complex, place-specific socioeconomic dynamics that drive property values.

**Fig 4:**
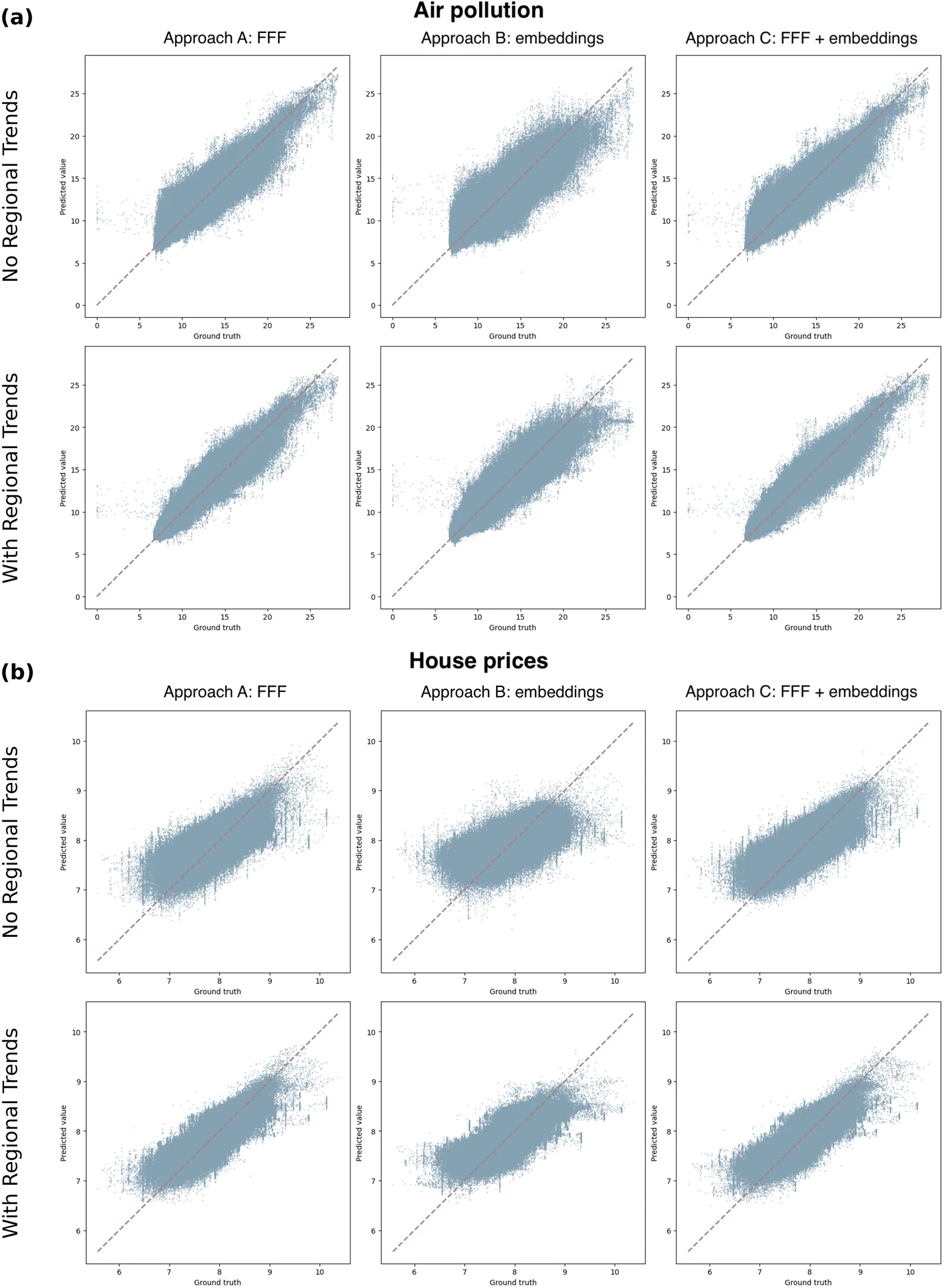
Predicted values against ground truth for approaches A, B and C. (a) Air pollution (top) and (b) house price indices (bottom); approaches A, B and C from left to right.

Comparing predictions against observed data across all methods (Fig 4) further underscored these insights: air pollution predictions closely aligned with observed values, whereas house price estimates showed greater variability. The marked improvement from including regional trends underscores the necessity of spatial context, especially in embedding-based approaches.

Taken together, our findings highlight a key insight: satellite image embeddings, powered by foundation models, can become competitive with traditional structured datasets — most clearly for air pollution — while significantly reducing data-collection costs and computational complexity. Their effectiveness, however, strongly depends on incorporating spatial context, and for house prices much of the gain attributable to embeddings is in fact coarse regional location. This emphasises that embeddings are best understood as a complement to, rather than a substitute for, traditional data.

## Discussion

Our analysis demonstrates that foundation model embeddings derived from satellite imagery provide a competitive alternative to traditional structured datasets for assessing urban inequalities. Although traditional sources, such as census and survey data, continue to offer robust and detailed information, satellite-based embeddings hold considerable promise, especially when rapid and flexible analyses are needed. Crucially, embedding-based models significantly benefit from the integration of regional context, which enhances their predictive capabilities by situating abstract visual information within broader spatial patterns.

Satellite embeddings performed particularly well in predicting air pollution across England. This is consistent with the understanding that while air pollution itself is not directly visible in imagery, the underlying infrastructure and land-use patterns contributing to pollution often are. Prior studies leveraging deep learning with satellite imagery to predict environmental variables typically required either very high-resolution imagery or substantial model fine-tuning [47–49]. Although foundation models have rarely been evaluated explicitly on environmental benchmarks such as air pollution, our results suggest that their embeddings nonetheless effectively capture meaningful predictive signals. A caveat applies to the interpretation of these gains. Because the SatlasPretrain backbone is pre-trained on Sentinel-2 imagery, part of the embeddings’ advantage may reflect domain alignment between the pre-training and evaluation data rather than a general property of foundation-model embeddings. Our results may therefore not transfer directly to embeddings derived from other sensors, resolutions, or pre-training distributions, and systematic benchmarking across alternative backbones (for example Prithvi [23] or SatMAE [24]) is an important direction for future work. More broadly, geospatial foundation models are advancing rapidly: the backbone used here is single-sensor (Sentinel-2 only), whereas more recent models are increasingly multi-sensor and multi-resolution, jointly encoding optical, radar and finer-resolution inputs (for example, DOFA [50]). Our off-the-shelf results are therefore best interpreted as a conservative lower bound on the performance achievable with image embeddings, likely to rise as such models mature.

In contrast, predicting house prices from imagery proved more challenging. The complexity of socioeconomic outcomes inherently limits predictions derived solely from visual information. House prices reflect nuanced factors such as wealth distribution, neighbourhood desirability, safety, and proximity to amenities—all of which involve intangible attributes not always clearly visible from medium-resolution overhead images. Previous studies using street-level imagery and night-time lights have successfully identified visual proxies linked to socioeconomic conditions, such as signs of property maintenance or degradation [36–39]. However, at Sentinel-2’s spatial resolution, these indicators may only be captured at a generalised scale, likely obscuring subtler visual cues related to property values. Our results confirm that while certain physical features indicative of neighbourhood desirability can be visually inferred, Sentinel-2 imagery is insufficient to capture the detailed local variations that significantly influence housing markets. The region-only baseline is consistent with, but does not on its own confirm, a target-visibility explanation: coarse location alone reaches $R^{2}\approx 0.48$ for house prices while the embeddings reach only 0.37, which would be expected if the relevant socioeconomic signal is only weakly observable at Sentinel-2’s ~10 m resolution. We caution, however, that our design does not isolate the mechanism: we cannot rule out that the embeddings themselves do not yet capture enough housing-relevant signal, and the two explanations are not mutually exclusive.

We stress that the comparison is between practical end-to-end pipelines rather than between the raw representational capacities of the two data types: FFF combines 59 curated variables with their spatial lags, while the embedding compresses image content into 128 dimensions, and the two differ markedly in preparation and processing cost. A key strength of our approach lies in the ease of implementation and relatively low technical complexity of using pre-trained embeddings. Unlike traditional neural network workflows, which typically require extensive fine-tuning, considerable computing resources, and advanced technical skills, pre-trained embeddings simplify the analytical process considerably. Our experience indicates that analysts with moderate data science capabilities can easily integrate these embeddings into predictive workflows, substantially lowering barriers for incorporating advanced geospatial AI into practical urban analysis [13]. Furthermore, producing these embeddings entails moderate computational costs, comparable to conventional structured data processing, especially considering the significant reduction in data-collection efforts.

These findings also underscore the potential of satellite imagery, and specifically foundation-model-derived embeddings, to address critical data gaps in urban research. Traditional data sources often lag behind the pace of urban change, providing only infrequent temporal snapshots and limited spatial detail. By contrast, satellite imagery offers regular, detailed updates, making it uniquely suited for timely monitoring of urban environments. Yet access to very high-resolution imagery remains expensive or restricted, presenting barriers for widespread adoption. Foundation models, by extracting meaningful features even from freely available medium-resolution imagery, substantially enhance the accessibility and utility of satellite-based analyses, particularly in resource-limited settings. Partnerships between public sector agencies, data providers, and open-data initiatives could further democratise these powerful analytical tools.

Our findings indicate considerable potential for scalability. While our analysis was focused on England, the methodologies we used can readily be extended globally, exploring additional indicators relevant to urban inequalities, such as green-space accessibility, public transportation availability, or localised environmental hazards. We note that England’s hexes are predominantly rural by land area (approximately 91% countryside or buffer, 58% in the countryside-agriculture signature and only ~0.2% dense-urban). This is a characteristic of the land surface rather than a sampling bias in the outcomes: the targets are populated throughout and a positive house price is available for 96–99% of hexes within every signature type. The five spatial folds are disjoint by H3 resolution-6 parent and each spans 24–26 of the 26 regions, so no region or settlement type is systematically excluded from training or testing. Expanding the scope of indicators tested would provide further insight into the flexibility and applicability of foundation model embeddings across diverse contexts. Moreover, systematically benchmarking foundational models across varied urban environments can guide researchers and policymakers in selecting the most effective tools for their particular analytical needs.

Ultimately, the greatest promise of foundation models may reside in their application to longitudinal analyses of urban inequality. Satellite embeddings offer a flexible and efficient means of capturing urban dynamics over time, enabling near-real-time monitoring of processes like gentrification, infrastructure development, or environmental degradation. Coupled with time-series analysis, such embeddings could support early detection of emerging inequalities, evaluation of policy impacts, and timely recalibration of urban interventions.

In summary, our study shows that satellite-based embeddings, while not yet surpassing the accuracy of traditional urban datasets, offer a powerful complementary tool due to their frequent updates, lower data-collection costs, and reduced computational complexity. Future improvements in embedding quality, increased availability of high-resolution satellite imagery, and the establishment of standardised evaluation frameworks will likely further enhance their practical applicability, contributing significantly to more equitable and sustainable urban futures.

## Conclusion

This paper aimed to systematically assess the predictive performance and practical utility of satellite image embeddings, generated by geospatial foundation models, compared to traditional structured geospatial data for capturing urban inequalities. By comparing these approaches across two critical indicators, air pollution and house prices, we demonstrated that while traditional, survey-based datasets remain highly effective, satellite-based embeddings offer competitive accuracy, particularly when enriched with regional spatial context. These results underscore the significant potential of foundation models for leveraging frequently updated, globally available satellite imagery, thereby offering a flexible, efficient complement to static, resource-intensive traditional datasets.

Rather than treating satellite embeddings and conventional structured data as substitutes, our findings indicate that they are most effectively viewed as complementary tools. Embeddings derived from foundation models capture substantial portions of the critical information traditionally obtained from censuses and administrative sources, thereby enabling more responsive and scalable urban analytics. Such timely analytical capability can empower policymakers and urban planners to monitor changes in urban conditions at unprecedented spatial and temporal resolutions.

Nevertheless, fully harnessing the potential of foundation models will require additional refinement and exploration. Future research directions include investigating higher-resolution satellite imagery, developing embeddings tailored to specific socioeconomic and environmental variables, and expanding the range of indicators analysed. As the ecosystem of geospatial artificial intelligence continues to mature, satellite-derived analytical methods will likely become increasingly indispensable, providing valuable insights for equitable and sustainable urban development and enabling innovative strategies to address complex urban challenges.

## Supporting information

S1 Fig Hexagonal masking of image tiles. From left to right: the first image tile represents the unmasked tile, the middle tile represents the H3 mask of that specific location, and the right mask is the summed mask over the whole dataset. Contains modified Copernicus Sentinel-2 data (2017–2018) from the global cloud-free composite of Corbane et al. [40].
(TIF)

S2 FigEmbedding network architecture.The 42×42 pixel images are fed into a pre-trained network, which consists of a Swin Transformer that outputs four feature maps. The feature maps are reduced with a Feature Pyramid Network (FPN) and summarised with a pooling layer into the final feature embedding vector.(TIF)

S3 Fig H3 resolution-3 hexagons over England. The H3 hexagons at resolution 3 plotted on top of a map of England. The England outline is derived from Office for National Statistics boundary data (Contains OS data © Crown copyright and database right, Open Government Licence v3.0).
(TIF)

S1 Table Best model conditions, air pollution model. XGBoost hyperparameters (maximum tree depth, number of estimators, and learning rate) selected for each approach (FFF, embeddings, and FFF + embeddings, each with and without regional context) for the air-pollution model.
(TIF)

S2 Table Best model conditions, house price model. XGBoost hyperparameters (maximum tree depth, number of estimators, and learning rate) selected for each approach for the house-price model.
(TIF)

S3 Table Predictor variables: population and workplace. Full list of the population and workplace predictor variables (features of form and function) used in the models.
(TIF)

S4 Table Predictor variables: CORINE land cover and morphometrics. Full list of the CORINE land-cover and morphometric predictor variables (features of form and function) used in the models.
(PDF)

S1 File Features of form and function: data sources and variable reduction. Detailed description of the four predictor-variable data sources (population, workplace, CORINE land cover, and Urban Grammar morphometrics), their harmonisation to Output Area level, and the correlation-based procedure used to reduce the variable set from 75 to 59 variables.
(PDF)
