# Supplementary material for: Is a (satellite) image worth a thousand data points? Comparing machine learning approaches to predict environmental and social inequalities in England
Source: PLoS One. 2026 Aug 21;21(8):e0356472. doi: 10.1371/journal.pone.0356472 (PMC13497279; doi:10.1371/journal.pone.0356472)
Supplement: Supplementary file 1 [file pone.0356472.s007.pdf]

S4 Table.

Predictor variables: CORINE land cover and morphometrics. Land-cover and morphometric features of form and function used in the models.

#	**Variable**
**CORINE land cover**	
10	Discontinuous urban fabric
11	Continuous urban fabric
12	Non-irrigated arable land
13	Industrial or commercial units
14	Green urban areas
15	Pastures
16	Sport and leisure facilities
**Morphometrics**	
17	Area of building
18	Courtyard area of building
19	Circular compactness of building
20	Corners of building
21	Squareness of building
22	Equivalent rectangular index of building
23	Centroid-corner mean distance of building
24	Centroid-corner distance deviation of building
25	Orientation of building
26	Area of ETC
27	Circular compactness of ETC
28	Equivalent rectangular index of ETC
29	Covered area ratio of ETC
30	Cell alignment of building
31	Alignment of neighbouring buildings
32	Mean distance between neighbouring buildings
33	Perimeter-weighted neighbours of ETC
34	Mean inter-building distance
35	Width of street profile
36	Width deviation of street profile
37	Openness of street profile
38	Length of street segment
39	Linearity of street segment
40	Mean segment length within 3 steps
41	Node degree of junction
42	Local proportion of 3-way intersections of street network
43	Local proportion of 4-way intersections of street network
44	Local proportion of cul-de-sacs of street network
45	Local closeness of street network
46	Local cul-de-sac length of street network
47	Square clustering of street network
48	Local degree weighted node density of street network
49	Street alignment of building
50	Area covered by edge-attached ETCs
51	Buildings per meter of street segment

#	**Variable**
52	Reached ETCs by neighbouring segments
53	Reached ETCs by tessellation contiguity
54	Area of enclosure
55	Circular compactness of enclosure
56	Equivalent rectangular index of enclosure
57	Orientation of enclosure
58	Perimeter-weighted neighbours of enclosure
59	Area-weighted ETCs of enclosure
