# Supplementary material for: Is a (satellite) image worth a thousand data points? Comparing machine learning approaches to predict environmental and social inequalities in England
Source: PLoS One. 2026 Aug 21;21(8):e0356472. doi: 10.1371/journal.pone.0356472 (PMC13497279; doi:10.1371/journal.pone.0356472)
Supplement: Supplementary file 2 [file pone.0356472.s008.pdf]

S1 File.

Features of form and function: data sources and variable reduction.

We assembled a comprehensive set of predictor variables from four distinct data sources, carefully harmonised to match the study's spatial and temporal scales.

Population data. We obtained mid-2020 population estimates at the Output Area (OA) level directly from the Office for National Statistics (ONS) Small Area Population Estimates. This dataset required no further preprocessing, as it already matches the required spatial granularity and is temporally consistent with other data sources reflecting the 2018–2020 period.

Workplace population data. Workplace population statistics were sourced from the 2011 Census, originally reported at Workplace Zone level. We aggregated industry categories into eight broad groups for analytical simplicity: (A, B, D, E) agriculture, energy, and water; (C) manufacturing; (F) construction; (G, I) distribution, hotels, and restaurants; (H, J) transport and communication; (K, L, M, N) financial, real estate, professional, and administrative activities; (O, P, Q) public administration, education, and health; and (R, S, T, U) other. These data were spatially interpolated from Workplace Zones to OA geometries to align with the population data.

Land cover. CORINE Land Cover (2018) classification data, initially provided as polygonal features, were interpolated to OAs, calculating the percentage coverage for each OA. We excluded invariant categories, retaining seven relevant land-cover classes: discontinuous urban fabric; continuous urban fabric; non-irrigated arable land; industrial or commercial units; green urban areas; pastures; and sport and leisure facilities.

Morphometrics. To quantify urban morphological characteristics, we used detailed measurements from the Urban Grammar project, which defines and characterises the built environment in quantitative terms. The original morphometric variables were provided at the scale of enclosed tessellation cells (ETCs) and interpolated to OA level, producing an extensive contextual dataset.

Variable selection and reduction. Initially, these datasets collectively yielded 75 explanatory variables. Recognising potential redundancy, we performed a systematic variable reduction based on correlation analyses. Specifically, we assessed Pearson and Spearman correlation coefficients between all variable pairs. Where both correlation measures exceeded an absolute value of 0.8, we retained only the most interpretable variable from each correlated pair. This process reduced the morphometric variable set by 16, leaving a refined total of 59 explanatory variables (listed in S3 Table and S4 Table).

Data sources. ONS Small Area Population Estimates (rebased mid-2012 to mid-2020), Office for National Statistics; CORINE Land Cover 2018, Copernicus Land Monitoring Service; Urban Grammar morphometrics (Fleischmann & Arribas-Bel).
